# Embryonic Heterogeneity of Smooth Muscle Cells in the Complex Mechanisms of Thoracic Aortic Aneurysms

**DOI:** 10.3390/genes13091618

**Published:** 2022-09-09

**Authors:** Sohei Ito, Hong S. Lu, Alan Daugherty, Hisashi Sawada

**Affiliations:** 1 Saha Cardiovascular Research Center, College of Medicine, University of Kentucky, Lexington, KY 40536, USA; 2 Saha Aortic Center, College of Medicine, University of Kentucky, Lexington, KY 40536, USA; 3 Department of Physiology, College of Medicine, University of Kentucky, Lexington, KY 40536, USA

**Keywords:** aorta, aneurysm, cardiac neural crest, second heart field, smooth muscle, thoracic aorta

## Abstract

Smooth muscle cells (SMCs) are the major cell type of the aortic wall and play a pivotal role in the pathophysiology of thoracic aortic aneurysms (TAAs). TAAs occur in a region-specific manner with the proximal region being a common location. In this region, SMCs are derived embryonically from either the cardiac neural crest or the second heart field. These cells of distinct origins reside in specific locations and exhibit different biological behaviors in the complex mechanism of TAAs. The purpose of this review is to enhance understanding of the embryonic heterogeneity of SMCs in the proximal thoracic aorta and their functions in TAAs.

## 1. Introduction

Thoracic aortic aneurysms (TAAs) are life-threatening diseases defined as a dilatation of the aortic wall in the thoracic region [1]. TAAs occur either sporadically or in association with a genetic condition, including mutations in *FBN1* (encoding fibrillin-1); *ACTA2* (encoding α-smooth muscle actin); *MYH11* (encoding myosin heavy chain 11); and genes of transforming growth factor (TGF)-β and its receptors [2,3,4,5,6,7,8]. Despite the heterogeneous causes, a common feature of TAAs is the regional specificity that aortic dilatations occur predominantly in the proximal region: the aortic root and ascending aorta [9,10]. For example, patients with Marfan syndrome (MFS) and Ehlers-Danlos syndrome exhibit TAA formation preferentially in the aortic root [11,12,13], and TAAs in Loeys-Dietz syndrome (LDS) and Turner syndrome occur in the aortic root and the ascending aorta [14,15,16,17]. Another example is that bicuspid aortic valve (BAV) leads to TAA formation in the ascending aorta [18]. Multiple mouse models mimic these regional specificities of TAAs. MFS and LDS mouse models have luminal dilatations in the proximal thoracic aorta [19,20,21]. TAAs induced by chronic angiotensin II infusion, representing sporadic TAAs, are located mainly in the ascending aorta [22,23]. Several mechanisms have been reported as a determinant of the regional specificity of TAAs, such as hemodynamic effects due to the complex blood flow [24], the nonuniformity of vascular components across the aorta [25], and embryonic heterogeneity of SMCs [26,27].

SMCs are the most abundant cell type of the aortic wall [28]. Aortic SMCs are derived embryonically from several origins: second heart field (SHF), cardiac neural crest (CNC), somite, and splanchnic mesoderm [29,30]. In the disease-prone proximal thoracic aorta, SMCs are derived from both the SHF and CNC [27,30,31,32,33]. In the past decade, multiple studies have uncovered disparate biological functions of SMCs between their embryonic origins and the pathophysiology of aortic diseases, including TAAs [32,33,34,35,36,37,38,39,40]. This review highlights publications investigating the role of SMC origins and discusses functional divergences of these origins in the development of TAAs.

## 2. Distributions of CNC- and SHF-Derived SMCs 

The CNC is composed of mesenchymal cells derived from the ectoderm [41], which migrates into pharyngeal arches and the outflow tract. The SHF is derived from the mesoderm that forms a part of the cardiac crescent and migrates into the heart tube [42,43]. SHF-derived cells in the heart tube constitute the right ventricle and the proximal thoracic aorta. While selected cells of these origins are differentiated into endothelial cells and fibroblasts, most CNC- and SHF-derived cells in the thoracic aorta are differentiated into SMCs [30].

**Figure 1:**
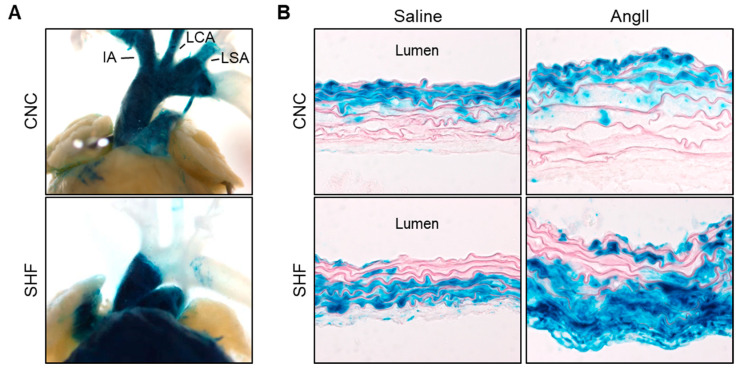
Embryonic origins of SMCs in the ascending aorta. Representative images of X-gal-stained aortic (**A**) tissues and (**B**) sections from *Wnt1-Cre* and *Mef2c-Cre* *ROSA26R*^LacZ^ mice. Blue color indicates the distribution of cells driven by either Cre. CNC indicates cardiac neural crest; SHF, second heart field; IA, innominate artery; LCA, left common carotid artery; LSA, left subclavian artery. Images are cited from [30,32] with permission from Wolters Kluwer Health (2022).

The distribution of CNC-derived SMCs in the proximal thoracic aorta was originally determined by Jiang et al. [44]. A fate-mapping study using mice expressing *Cre* driven by a *Wnt1* promoter revealed that CNC-derived SMCs populate the thoracic aorta from the ascending aorta and throughout the aortic arch (Figure 1A). This distinct distribution has been validated by multiple studies [30,33,45,46,47], and *Wnt1-Cre* is now a common promoter in studies of CNC-derived SMCs. As shown in cross sections of aortic tissue, CNC-derived SMCs are distributed in the whole media of the posterior curvature of the ascending aorta, but only in the inner media of the anterior curvature (Figure 1B) [30].

SHF-derived cells were initially mapped using avian systems [48]. A fluorescent dye was microinjected into the SHF of chick embryos and the stained cells were tracked. SHF-derived cells migrate into the myocardial outflow myocardium and the outflow tract. These findings were confirmed by subsequent studies using fate mapping in mouse models [30,33,34,49]. Several promoters are available for *Cre* to track SHF-derived cells in mice: *Nkx2.5*, *Mef2c*, and *Islet1*. Despite some disparities of distributions in the myocardium, these promoters demonstrate consistent distributions in the proximal thoracic aorta that SHF-derived cells populate the aortic root and ascending aorta [30,33,34,49] (Figure 1A). Unlike CNC-derived cells, SHF-derived cells do not extend to the aortic arch. SHF-derived SMCs also have a unique distribution in the media [30]. SHF-derived SMCs are present mainly in the outer media of the ascending aorta (Figure 1B). Thus, the proximal thoracic aorta contains overlapping SMCs from both CNC and SHF origins, and these origins show a spatially distinct distribution.

In humans, aortic medial pathologies, such as a loss of SMCs and collagen deposition, exhibit a gradient across the media that increases from the luminal to the adventitial aspects [23,32]. Aortic dissection occurs preferentially in the outer third of the aortic media [50]. Multiple TAA mouse models also exhibit outer media-dominant pathologies, such as thickening and hemorrhage (Figure 1B) [32,51,52,53]. Thus, medial pathologies show a gradient toward the outer medial aspect in human and mouse TAAs. The gradient of medial pathologies in TAAs corresponds to the distribution of embryologic origins of SMCs that has been shown in mouse studies, indicating that SMCs of different embryonic origins have different functions in the pathophysiology of TAAs.

## 3. Functional Differences between Embryonic Origins of SMCs in Development of TAAs

In the past decade, multiple studies have uncovered functional differences between CNC- and SHF-derived SMCs in maintaining aortic structure and function (Table 1 and Table 2) [32,33,34,35,36,37,38].

### 3.1. Marfan Syndrome (MFS)

MFS is a multisystem disorder resulting from mutations in *FBN1*, encoding fibrillin-1 [11]. TAAs are a devastating manifestation of this syndrome. There is evidence that aortic TGF-β is upregulated in a mode that corresponds with luminal dilatations in MFS [15,55]. The impact of SMC origins on the dysregulation of TGF-β signaling has been investigated using induced pluripotent stem cells (iPSCs) [36]. iPSCs were generated from either patients with MFS or control subjects. Subsequently, iPSCs were differentiated into lateral mesoderm-, paraxial mesoderm-, and neural crest-derived SMCs. Compared to control subjects, the abundance of TGF-β ligands was increased in MFS-SMCs derived from the neural crest, but not from other origins (Table 2). In addition, neural crest-derived MFS-SMCs exhibited severe abnormal organization of extracellular microfibrils. These findings suggest that neural crest-derived SMCs are more susceptible to *FBN1* mutations than SMCs from other origins.

The NOTCH1 signaling pathway is important for cardiovascular development and aortic integrity [56,57]. The heterozygous deletion of NOTCH1 in pan-SMCs augmented luminal dilatations in the aortic sinus and disrupted the extracellular matrix in *Fbn1* haploinsufficient (*Fbn1^C1041G/+^*) mice [37]. Of note, the heterozygous deletion of NOTCH1 in SHF-, but not CNC-, derived cells had a tendency to recapitulate these aortic pathologies (*p* = 0.08, Table 1). In contrast to the human iPSC data, mouse models revealed a potential role of SHF-derived cells in TAA formation of MFS mice through NOTCH1-mediated mechanisms.

Single-cell RNA sequencing (scRNAseq) using a fate-mapping strategy in mice enables the precise and unbiased determination of biological differences of SMCs between origins. A recent study by Pedroza et al. performed scRNAseq in the proximal thoracic aorta of *Fbn1* haploinsufficient mice with tdTomato reporter driven by *Nkx2.5* [39]. Cells were selected based on tdTomato signals in SHF-derived cells, and transcriptomes were compared between origins. CNC-derived SMCs displayed a chondrogenic phenotype, whereas SHF-derived SMCs had abundant multiple collagen genes (Table 2). In addition, the transcriptional activity of TWIST1, a mediator of pathologic fibrosis, was enhanced in SHF-derived SMCs compared to CNC-derived SMCs. In MFS, genetic mutations on *Fbn1* lead to multiple functional alterations of SMCs in an embryonic origin-specific manner. However, its impact on TAA formation is not fully understood. Further in vivo studies with genetic manipulations in each origin would be helpful to understand the molecular basis of embryonic differences in the pathophysiology of TAAs in MFS.

### 3.2. Loeys-Dietz Syndrome (LDS)

Patients with LDS have an aggressive TAA formation caused by mutations in genes encoding either type 1 or 2 TGF-β receptors [14] and the downstream pathways. Although TGF-β receptors are obligatory for TGF-β signaling, LDS exhibits characteristics that have been interpreted as overactivated TGF-β pathways including increased SMAD2/3 phosphorylation in the aorta [21]. In vitro experiments using iPSCs from LDS patients with gene mutations on *TGFBR1* (*TGFBR1^A230T^*) revealed that *TGFBR1* mutation downregulates SMAD3 phosphorylation in SHF-derived SMCs, whereas it is not altered in CNC-derived SMCs (Table 2) [38]. The same group also investigated the impact of lineage-specific *SMAD3* mutation on aortic TGF-β signaling activity [35]. Aortic SMAD2 phosphorylation was not changed by *SMAD3* mutations in SHF-derived SMCs, but *SMAD3* mutations increased SMAD2 phosphorylation in CNC-derived SMCs (Table 2). Collectively, CNC- and SHF-derived SMCs demonstrate different responses to different mutations on TGF-β signaling molecules in LDS.

Mice with heterozygous missense mutation on *Tgrbr1* (*Tgrbr1*^M318R/+^) develop aortic root aneurysms and medial disruptions that mimic many facets of aortic pathologies in patients with LDS [21]. Distinct properties of CNC- and SHF-derived SMCs have also been observed in this LDS mouse model [33]. In vitro experiments defined that SHF-derived SMCs show impaired SMAD2/3 activation in response to TGF-β stimulation and an increased abundance of TGF-β ligands. In contrast, CNC-derived SMCs preserve TGF-β signaling potential without the alteration of TGF-β abundance. Of interest, aortic root dilatations are ameliorated by SMAD2 deletion in cells derived from the CNC, but not SHF in mice (Table 1). These findings indicate a critical role of CNC-derived SMCs in the development of TAAs through TGF-β signaling.

The constitutive deletion of TGFBR2 in pan-SMCs causes cardiovascular defects and embryonic lethality in mice [58]. Consistent with these phenotypes, TGFBR2 deletion in either CNC- or SHF-derived cells also causes vascular malformation (Table 1) [32,54]. CNC-specific TGFBR2 deficient mice die in the early postnatal phase with persistent truncus arteiosus and craniofacial defects [54]. TGFBR2 deletion in SHF-derived cells induces prenatal death around E11.5–12.5 with dilatation of the outflow tract and retroperitoneal hemorrhage [32]. Thus, both CNC and SHF origins play an important role in aortic development through TGFBR2.

There is compelling evidence that the renin-angiotensin system exerts a pivotal role in the development of TAAs [59]. Losartan, an angiotensin receptor blocker, ameliorates aneurysm formation in multiple mouse models, including LDS [21,60,61]. In *Tgrbr1*^M318R/+^ LDS mice, mRNA abundance of *Agtr1a* encoding angiotensin II (AngII) type 1a receptor is increased in SHF-derived SMCs, but not in CNC-derived cells [33]. In agreement, in vitro experiments revealed that AngII stimulation upregulates *Tgfb1* and *Tgfb3* mRNA in SHF-derived cells, which is suppressed by losartan. However, in vivo studies using LDS mice demonstrated that *Agtr1a* deletion in SHF-derived cells results in only a modest reduction in aortic dilatations (Table 1) [40]. Since TAA formation in LDS mice is attenuated remarkably by either pharmacological inhibition of AT1 receptors or whole-body *Agtr1a* genetic deficiency [21,40], it would be interesting to investigate the impact of *Agtr1a* in other cell types, including CNC-derived SMCs, on AngII-mediated mechanisms of TAAs in LDS.

TAAs are present in both MFS and LDS. However, the region prone to TAA formation differs between the two syndromes. Aortic dilatations in MFS are located primarily in the aortic root, whereas LDS displays aneurysm formation in both the aortic root and the ascending aorta [11,12,14,15]. Of note, the population of SMC origins is different between the aortic root and ascending aorta. The aortic root is predominantly populated with SHF-derived SMCs, while the ascending aorta is composed of both CNC- and SHF-derived SMCs [30]. SMCs show functional differences between origins (Table 1 and Table 2). Thus, the difference in SMC populations may contribute to the regional specificity of TAAs in MFS and LDS.

### 3.3. Angiotensin II-Mediated TAAs

AngII infusion leads to aortopathies, including luminal dilatation and medial thickening, in the ascending aorta of mice [22,23,32,62,63]. SMC-specific deletion of low-density lipoprotein receptor-related protein 1 (LRP1) that plays a critical role in extracellular matrix maturation augments AngII-induced aortopathies [64,65]. Of note, SHF-specific LRP1 deletion recapitulates the ascending aortic pathologies in AngII-infused mice with LRP1 deletion in pan-SMCs (Table 1) [32]. These data suggest that SHF-derived cells exert a critical role in AngII-induced TAA formation. scRNAseq using *Mef2c-Cre ROSA26R^mTmG^* mice demonstrated that a short-interval of AngII infusion decreased mRNA abundance of TGF-β receptors (*Tgfbr1*, *Tgfbr2*) in SHF-derived SMCs prior to TAA formation [32]. Thus, AngII compromises the TGF-β signaling pathway in SHF-derived SMCs that is vital in maintaining the aortic integrity. In contrast, there are no publications describing LRP1 in CNC-derived cells. In addition, transcriptomic alteration in CNC-derived cells by AngII infusion has not been determined. Further study, including scRNAseq, is desirable to uncover the role of CNC-derived cells in AngII-mediated aortopathy formation.

AngII-induced medial thickening shows a transmedial gradient that is dominant in the outer media. This pathological gradient is consistent with the distribution of SHF-derived SMCs (Figure 1B) [27,32], suggesting the susceptibility of SHF-derived SMCs to AngII-induced pathologies. Since the gradient of medial thickening is observed in other TAA mouse models and human TAAs [23,32,51,52,53], SHF-derived SMCs may play an important role in the pathophysiology of TAAs. It will be fascinating to investigate the molecular mechanisms of how SHF-derived cells are involved in the transmedial gradient of medial pathologies in TAAs. 

### 3.4. Other Aortic Diseases

Elastin is the major extracellular component of the aorta and a key determinant factor for aortic resilience [28]. Numerous studies have reported elastic fiber disruption as a key structural alteration in TAAs [66]. Nevertheless, genetic deletion of elastin did not cause TAA formation. Whole-body elastin deletion led to aortic stenosis by neointimal hyperplasia of SMCs in the proximal thoracic aorta [31,67,68]. Of note, elastin deletion in cells from either CNC- or SHF-derived cells also developed neointimal SMC hyperplasia (Table 1) [34]. Interestingly, the aortic neointima, despite being adjacent to the CNC-derived cells, is predominantly composed of SHF-lineage cells. 

CNC- and SHF-derived cells contribute to aortic valve development in addition to the proximal thoracic aorta [69]. Lineage tracking studies using *Wnt1* and *Nkx2.5* promotors revealed that right- and left-coronary leaflets are primarily composed of CNC-derived cells, whereas the non-coronary leaflet is derived from the SHF. These origins are associated with the pathophysiology of BAV [70]. CNC-specific *Krox20* deletion leads to BAV with the fusion of non- and right-coronary leaflets [71]. Although lesions of valve fusion and the incidence of BAV vary by genes, SHF-specific deficiency of *Gata6*, *Vangl2*, *Jag1*, or *Mib1* also displays BAV in mice [72,73,74].

Vascular Ehlers-Danlos syndrome (vEDS) is an autosomal dominant disorder caused by genetic mutations in *COL3A1* [75]. Similar to MFS and LDS, vEDS also shows the regional specificity of TAAs that the proximal thoracic region is dominant for aneurysm formation [13,76]. Although preclinical investigation of vEDS was restricted by the lack of animal models, a recent study established a mouse model that mimics multiple facets of vEDS [77]. Thus, it would be interesting to explore the contribution of SMC origins to vEDS-induced TAAs. 

## 4. Summary

CNC- and SHF-derived SMCs reside in distinct locations of the proximal thoracic aorta. Multiple studies have uncovered embryonic origin-specific mechanisms in aortic diseases, including TAAs. However, CNC- and SHF-derived cells demonstrate distinct properties in different regions and diseases, which has painted a complex landscape for origin-specific mechanisms in aortic diseases. Since TAAs are mediated by complex mechanisms, including the alterations in the extracellular matrix, mechano-transduction, and SMC functions [78,79,80,81], it is important to continue efforts to understand the divergent behaviors of embryonic origins in the pathophysiology of TAAs.

## Figures and Tables

**Table 1:** Aortic phenotypes caused by genetic manipulations in either SHF- or CNC-derived cells in mice.

Gene	Mouse Model	Aortic Phenotypes		Ref.
		CNC	SHF	
*Notch1*	*Fbn1^C1041G/+^*	TAA ↔	TAA ↑ (trend, *p* = 0.08)	[37]
*Fbn1*	*Fbn1^C1041G/+^*	Chondrogenic	Collagenic	[39]
*Tgfbr2*	Spontaneous	Persistent truncus arteriosus	Outflow tract dilatation	[32,54]
*Smad2*	*Tgfbr1^M318R/+^*	TAA ↓	TAA ↔	[33]
*Agtr1a*	*Tgfbr1^M318R/+^*	N/D	TAA ↓ (modestly)	[40]
*Lrp1*	AngII infusion	N/D	TAA ↑	[32]
*Eln*	Spontaneous	Neointimal hyperplasia	Neointimal hyperplasia	[34]

N/D indicates not determined; ↑, augmented; ↔, not changed; ↓, suppressed.

**Table 2:** TGF-β-related phenotypes in SHF- and CNC-derived SMCs generated from human iPSCs.

Model	Experiment	Phenotype		Ref.
		CNC	SHF	
iPSCs generated from MFS patients	In vitro	TGF-β1 ↑	TGF-β1 ↔	[36]
iPSCs generated from LDS patients	In vitro	pSMAD3 ↔	pSMAD3 ↓	[38]
iPSCs with LoF mutations on SMAD3 generated from a healthy donor	In vitro	pSMAD2 ↑	pSMAD2 ↔	[35]

LoF indicates loss of function; ↑, augmented; ↔, not changed; ↓, suppressed.
